# Mash1-dependent Notch Signaling Pathway Regulates GABAergic Neuron-Like Differentiation from Bone Marrow-Derived Mesenchymal Stem Cells

**DOI:** 10.14336/AD.2016.1018

**Published:** 2017-05-02

**Authors:** Qianfa Long, Qiang Luo, Kai Wang, Adrian Bates, Ashok K. Shetty

**Affiliations:** ^1^Department of Neurosurgery, Xi’an Central Hospital, Xi’an Jiao Tong University School of Medicine, Xi’an 710003, China; ^2^Institute for Regenerative Medicine, Texas A&M Health Science Center College of Medicine at Scott & White, Temple and College Station, Texas, 76502, USA; ^3^Department of Neurosurgery, Qingdao 401 Hospital of PLA, Qingdao 266071, China; ^4^Research Service, Olin E. Teague Veterans’ Medical Center, CTVHCS, Temple, Texas, USA

**Keywords:** Notch signaling pathway, bone marrow-derived mesenchymal stem cells, mammalian achaete scute homolog-1, hairy and enhancer of split-1, GABAergic neuron-like cells

## Abstract

GABAergic neuronal cell grafting has promise for treating a multitude of neurological disorders including epilepsy, age-related memory dysfunction, Alzheimer’s disease and schizophrenia. However, identification of an unlimited source of GABAergic cells is critical for advancing such therapies. Our previous study implied that reprogramming of bone marrow-derived mesenchymal stem cells (BMSCs) through overexpression of the Achaete-scute homolog 1 (Ascl1, also called Mash1) could generate GABAergic neuron-like cells. Here, we investigated mechanisms underlying the conversion of BMSCs into GABAergic cells. We inhibited γ-secretase (an enzyme that activates Notch signaling) with N-[N-(3,5-difluorophenacetyl)-L-alanyl]-S-phenylglycine t-butyl ester (DAPT) or manipulated the expression of Notch signaling components such as the *recombination signal binding protein for immunoglobulin kappa J region* (RBPJ), *hairy and enhancer of split-1* (Hes1) or Mash1. We demonstrate that inhibition of γ-secretase through DAPT down-regulates RBPJ and Hes1, up-regulates Mash1 and results in an enhanced differentiation of BMSCs into GABAergic cells. On the other hand, RBPJ knockdown in BMSCs has no effect on Mash1 gene expression whereas Hes1 knockdown increases the expression of Mash1. Transduction of Mash1 in BMSCs also increases the expression of Hes1 but not RBPJ. Moreover, increased GABAergic differentiation in BMSCs occurs with concurrent Mash1 overexpression and Hes1-silencing. Thus, the Mash1-dependent Notch signaling pathway regulates GABAergic neuron-like differentiation of BMSCs. These results also suggest that genetic engineering of BMSCs is a useful avenue for obtaining GABAergic neuron-like donor cells for the treatment of neurological disorders.

Gamma-amino butyric acid (GABA) is a chief inhibitory neurotransmitter that plays a principal role in reducing the neuronal excitability and synchronizing the firing of projection neuron ensembles in the mammalian central nervous system (CNS). Neurons that produce the neurotransmitter GABA are called GABAergic interneurons [1]. Loss or dysfunction of GABAergic interneurons is observed in a variety of disorders including epilepsy, age-related memory dysfunction, Alzheimer’s disease, schizophrenia, neuropathic pain and Parkinson’s disease [2]. Therefore, transplantation of GABAergic neuronal precursor cells into specific brain regions may be useful for treating these disorders. It has been shown that grafted GABAergic interneuron precursors survive and migrate in the host CNS, integrate into the circuitry, and exert therapeutic effects in multiple disease models. [1,3-5]. Namely, grafting of GABAergic precursor cells into the hippocampus in a mouse model of temporal lobe epilepsy reduced the extent of spontaneous seizures and abnormal behavior [3]; transplantation of GABAergic cells into the spinal cord reversed mechanical hypersensitivity in a neuropathic pain model [4]; and introduction of new GABAergic cells into the limbic cortex in a hippocampal disinhibition model reversed psychosis-relevant features [6]. Despite these exciting results, the clinical application of GABAergic precursor cell therapy is hampered by both difficulties in obtaining a sufficient amount of donor cells and ethical issues associated with the use of GABAergic precursor cells from fetal brains. From these perspectives, mesenchymal stem cells (MSCs) and induced pluripotent stem cells (iPSC) appear ideal as a starting material for obtaining large numbers of GABAergic precursor cells [7-9].

Cells induced from MSCs are commonly referred to as GABAergic neuron-like cells instead of full-fledged GABAergic neurons because they have been typified mostly through the occurrence of biomarkers such as GABA and GABA-synthesizing enzymes, a morphology showing soma and dendrite-like features [8, 10, 11]. Specific neuronal parameters such as synapse formation through axons, generation of action potentials and neurotransmission remain to be fully demonstrated in such cells [12]. Nonetheless, bone marrow-derived mesenchymal stem cells (BMSCs) have received significant attention due to their amenability for expansion in culture and minimal immunogenicity [13-15]. These cells also have the capability to differentiate into GABAergic neuron-like cells when genetically engineered or treated with a cocktail conditioned medium [7, 10, 11]. Furthermore, previous studies have shown that GABAergic neuron-like cells derived from BMSCs express GABA and GABA_A_ receptors following transplantation into the brain in a cerebral infarct model [16, 17]. These results have supported BMSCs as a potential source of GABA-synthesizing cells for grafting in neurological disorders. Moreover, a previous study in our laboratory demonstrated that an enhanced GABAergic neuron-like differentiation of BMSCs could be obtained *in vitro* through overexpression of the mammalian achaete scute homolog-1 (Ascl1, also called Mash1) gene [11]. Mash1 is a basic helix-loop-helix (bHLH) transcription factor, which has an important role in Notch signaling and the differentiation of GABAergic neurons [18, 19]. The Notch signaling pathway controls the fate-choice decision by stem cells, including MSCs. Multiple studies have shown that Notch activation maintains stemness whereas Notch inhibition causes precocious neuronal differentiation and progenitor cell depletion [20-23]. However, it is unknown whether and how Notch signaling is involved in the differentiation of MSCs into GABAergic cells.

The trans-membrane protein Notch is cleaved by γ-secretase through interactions with Notch ligands such as *Delta-like1* (Dll1) and *Jagged1* (Jag1), which leads to the release of *Notch intracellular domain* (NICD) from the plasma membrane. NICD translocates into the nucleus and forms a complex with the DNA binding protein *transcription inhibitor-recombination signal binding protein for immunoglobulin kappa J region* (RBPJ). This binding causes a conformational change that leads to the expression of downstream target genes such as *hairy and enhancer of split-1* (Hes1), which represses differentiation determination genes such as Mash1 by binding to the N box-related sequence of the Mash1 promoter [20, 22, 24] and maintains stemness of cells. Additionally, Mash1 can induce the expression of Notch ligands (e.g. Dll1), followed by activation of Notch signaling and upregulation of Hes1 expression through the RBPJ gene and Hes1 promoter combination, ultimately shaping a balance of Notch signaling in controlling cell proliferation and differentiation [25]. RBPJ-Hes1-Mash1 signaling, especially, as a part of the complicated Notch signaling system, is required for the specification of GABAergic neurons from neural stem/progenitor cells (NSCs) [26, 27]. Many studies have suggested that modulation of this signaling through RBPJ inhibition, Hes1 silencing, or Mash1 overexpression, can initiate and enhance the differentiation of NSCs into GABAergic neurons. Conversely, the overexpression of RBPJ and Hes1, or silencing of Mash1 results in the maintenance of stemness or progenitor status [26, 28-30]. In addition, these modulators (RBPJ, Hes1 and Mash1) have been shown to participate in the determination of the fate of MSCs including their differentiation into neuron-like cells [11, 21, 23, 31]. Therefore, in this study, we assessed which components of the Notch signaling pathway (RBPJ-Hes1-Mash1) regulate the GABAergic neuron-like differentiation of BMSCs *in vitro*.

## MATERIALS AND METHODS

### BMSCs isolation and characterization

Two to three week old male Sprague-Dawley (SD, n = 8) rats (weight 40-55 g) were purchased from the Experimental Animal Center of Xi’an Jiaotong University. All procedures on animals were performed in accordance with the guidelines for the Care and Use of Laboratory Animals of the National Institute of Health. BMSCs were isolated from donor rats as detailed in our previous study [11, 32]. Briefly, rats were sacrificed by exposure to carbon dioxide gas, and bone marrow was collected from sterile femurs and tibias. Mononuclear cells were harvested by density gradient centrifugation (400*g*, 25 minutes) with Histopaque®-1077 (Sigma, 10771, CA, USA) and viability of cells was evaluated using trypan blue exclusion test. For culturing, 60-100 viable cells/cm^2^ were plated onto 25 cm^2^ flasks in a complete medium comprising α-MEM (HyClone, UT, USA) and 10% fetal bovine serum (FBS) (Gibco BRL, MD, USA) (37°C, 5% CO_2_). The medium was changed every 2 days and cells were passaged with 0.25 % trypsin (containing 0.02 % EDTA) treatment when the adherent cells reached 70-80 % confluence. BMSCs from passage numbers 3-5 were characterized with flow cytometry. Primary antibodies such as rabbit polyclonal anti-CD105, CD90, CD73, CD45, CD34 or CD11b (1:100 dilution) (Bioss, Wuhan, CHN) and Alexa Fluor 488 goat anti-rabbit IgG (1:500) (Invitrogen, A-21206, CA, USA) were used to detect the surface antigens of BMSCs. At least three cell culture samples were evaluated on an FACS Calibur instrument (Becton Dickinson) and the data were analyzed using Cell Quest software (Becton Dickinson). Multi-potency of BMSCs was tested by StemPro® Osteogenesis (Gibco, A1007201, MD, USA), Chondrogenesis (Gibco, A1007101, MD, USA) or Adipogenesis (Gibco, A1007001, MD, USA) differentiation Kits (37 °C, 5% CO_2_) according to the instructions, and then the specific staining of alizarin red, toluidine blue or oil red O (Sigma, CA, USA) [33].

**Table 1:** Primer sequences for Notch signaling and GABAergic neuron marker

Gene name	Direction	Sequence of Primer (5′ - 3′)	Length (mer)	GC %	Tm (°C)	Gene ID (Gene Bank)
RBPJ	Forward	TTGCTTACCTTCAGGCGTGTG	21	52.4	63.5	679028
	Reverse	GCCCAATGAGTCTGCTGCAA	20	55	64.8	
Mash1	Forward	AGGCCCTACTGGGAATGGA	19	57.9	61.8	64186
	Reverse	CCCTGTTGCTGAGAACATTGA	21	47.6	61.2	
Hes1	Forward	GGAGAGGCTGCCAAGGTTTT	20	60	64.9	29577
	Reverse	AGGCGACACTGCGTTAGGA	19	52.2	64.5	
NeuN	Forward	AAGCTGAATGGGACGATCGTAGAG	24	50	65	287847
	Reverse	GCATAGAATTCGGGCCCATAGAC	23	52.2	64.9	
GAD67	Forward	GCTGGAAGGCATGGAAGGTTTTA	23	50	64.4	24379
	Reverse	AATATCCCATCACCATCTTTATTTGACC	28	52.2	63.8	
β-3 tubulin	Forward	CTCAGGGGCCTTTGGACATC	20	51.4	64.5	246118
	Reverse	CAGGCAGTCGCAGTTTTCAC	20	52.2	62	
NF-H	Forward	CTCCCAAAAATTCCCTCCATA	21	57	63.5	24587
	Reverse	CTGTCACTCCTTCCGTCACC	20	54.5	65.2	
GAPDH	Forward	GGCACAGTCAAGGCTGAGAATG	22	54.5	64.4	24383
	Reverse	ATGGTGGTGAAGACGCCAGTA	21	52.4	62.8	

### Plasmids, lentivirus production, and transduction

To achieve Mash1 gene overexpression, the DNA fragment of the Rat Mash1 gene was amplified by reverse transcription polymerase chain reaction (RT-PCR) with the primers as forward 5′-AGG TCG ACT CTA GAG GAT CCC GCC ACC ATG GAG AGC TCT GGC AAG ATG-3′ and reverse 5′-AGT CCA TGG TGG CGA CCG GGA ACC AGT TGG TAA AGT CCA G-3′. pGC-FU-Mash1 plasmid was produced by GeneChem (Shanghai, China) and lentivirus vector expression procedures were performed as described in our previous study [11]. BMSCs were then transduced with the overexpression of Mash1 (Mash1+BMSCs), by using transfected 293 T cells. For RBPJ or Hes1 silencing, BMSCs which reached approximately 50% confluence in a 12-well plate were replaced with Polybrene (Santa Cruz Biotechnology, Inc., sc-134220, CA, USA) media mixture, followed by infection with RBPJ (Santa Cruz Biotechnology, Inc., sc-270318-v, CA, USA) or Hes1 (Santa Cruz Biotechnology, Inc., sc-270146-v, CA, USA) shRNA lentiviral particles (these primers were provided by the company) overnight (37°C, 5% CO_2_). Then, puromycin dihydrochloride (Sigma, P9620, CA, USA) was used to select the stable clones expressing the RBPJ or Hes1 shRNA. Following this, RBPJ silenced BMSCs (RBPJ-BMSCs) or Hes1 silenced BMSCs (Hes1-BMSCs) were expanded for subsequent experiments. Meanwhile, BMSCs were transduced with copGFP control lentiviral particles (Santa Cruz Biotechnology, Inc., sc-108084, CA, USA) (Lv-con-BMSCs, control group) as in the above procedures. Quantitative real time PCR (qRT-PCR) (primer sequences listed in Table 1) and western blots were employed to confirm the signaling expression in the genetically engineered BMSCs.

### DAPT treatment and GABAergic induction

To indirectly inhibit the Notch signaling of BMSCs, 1 µM N-(3, 5-difluorophenylacetyl-L-alanyl)]-S-phenylglycine t-butylester (DAPT) (Sigma, D5942, CA, USA) was used as previously reported [31]. For GABAergic cell induction, different cell samples including BMSCs, DAPT treated BMSCs (DAPT+BMSCs), RBPJ-BMSCs, Hes1-BMSCs, Mash1+BMSCs or Lv-co-BMSCs were induced using a cocktail medium conditioned with B-27® Serum-Free Supplement (Gibco™, 17504044, NY, USA), 1 mM N5, O2′-dibutyryladenosine 3′:5′-cyclic monophosphate (Bt2cAMP) (Sigma, D0627, CA, USA) and 1 μM all-trans-retinoic acid (ATRA) (Sigma, R2500, CA, USA) in Neurobasal® Medium (Gibco™, 21103-049, NY, USA) for 1 week as previously described [11]. Conditioned medium was changed every day.

### Immunofluorescence

Induced cell samples were set on poly-L-lysine-coated confocal culture plates and fixed with 95 % ethanol for 30 min. Samples were then permeabilized with 0.1% TritonX-100 for 5 min, followed by blocking with 1% BSA for 30 min. Afterwards, primary antibodies including rabbit polyclonal anti-neurofilament, heavy polypeptide (NF-H, 1:500) (ThermoFisher, PA1-10016, NY, USA) / mouse monoclonal anti-GAD67 (1:50) (Abcam, ab26116, MA, USA), rabbit monoclonal anti-NeuN (1:100) (Millipore, MABN140, MA, USA) / mouse anti-GAD67 (1:50), or rabbit monoclonal anti-Class III β-tubulin (β-3 tubulin, 1:200) (Cell Signaling, 5568, MA, USA), mouse anti-GAD67 (1:50) were used for incubation overnight at 4 °C. Secondary antibodies such as Alexa Fluor 488 goat anti-rabbit IgG (1:500) and Alexa Fluor 594 goat anti-mouse IgG (1:500) (Invitrogen, A-21203, NY, USA) were applied for 2.5 h, then mounted using 4', 6-diamidino-2-phenylindole (DAPI, 1:1000, Sigma, 32670, CA, USA). Cultures processed without the use of the primary antibodies served as negative controls. Immunofluorescent images were analyzed using a confocal microscope (Olympus, FV10-ASW, JPN). Cells expressing GAD67 with NeuN, β-3 tubulin or NF-H dual immunofluorescence were identified and examined to confirm the GABAergic differentiation of BMSCs. The percentage of cells positive for both GAD67 and NeuN was quantified from five independent cultures.

### Protein preparation and western blotting

Pre-induced cell samples were examined for Notch signaling variation and induced cell samples were used for GABAergic neuron-like differentiation. These cell samples were treated with lysis buffer supplemented with phosphatase inhibitor cocktail (Sigma, P2850, CA, USA) and centrifuged (12000 rpm × 30min, 4 °C). Protein concentration was assessed using a BCA Protein Assay Kit (Pierce™, 23225, NY, USA), samples were recovered by electrophoresis (110V, 40min) on 10% SDS-PAGE gels and transferred to nitrocellulose membranes (ThermoFisher, 88024, CA, USA). Membranes were blocked in 5% non-fat milk at room temperature for 2 h. For Notch signaling examination, the pre-induced cell samples were incubated with rabbit monoclonal anti-Mash1 (1:500) (BD Pharmingen™, 556604, NY, USA), rabbit monoclonal anti-Hes1 (1:2000) (Cell Signaling, D6P2U, PA, USA), rabbit monoclonal anti-RBPJ (Cell Signaling, D10A4, PA, USA), or mouse monoclonal anti- β-actin (1:2000) (Abcam, ab14128, CA, USA). For GABAergic neuron-like differentiation detection, the induced cell samples were incubated with rabbit anti-NF-H, (1:2000), mouse anti-GAD67 (1:500), rabbit anti-NeuN (1:2000), rabbit anti-β-3 tubulin (1:2000), or mouse monoclonal anti-β-actin (1:2000) overnight at 4°C. Afterwards, HRP-conjugated secondary antibodies (1:5000) (Santa Cruz Biotechnology, Inc., sc-2370 or sc-2371, CA, USA) were used to probe these specific antibodies for 1 h at room temperature. Subsequently, the reactions were visualized using ECL Western blotting detection (Amersham Life Science, Piscataway, NJ, USA) and luminal excitation was imaged on X-ray film. For each sample, at least three independent experiments were performed. Normalization was based on the Gauss Model Trace of β-actin and analyzed using Quantity One 4.6.2 software.

**Figure 1.**
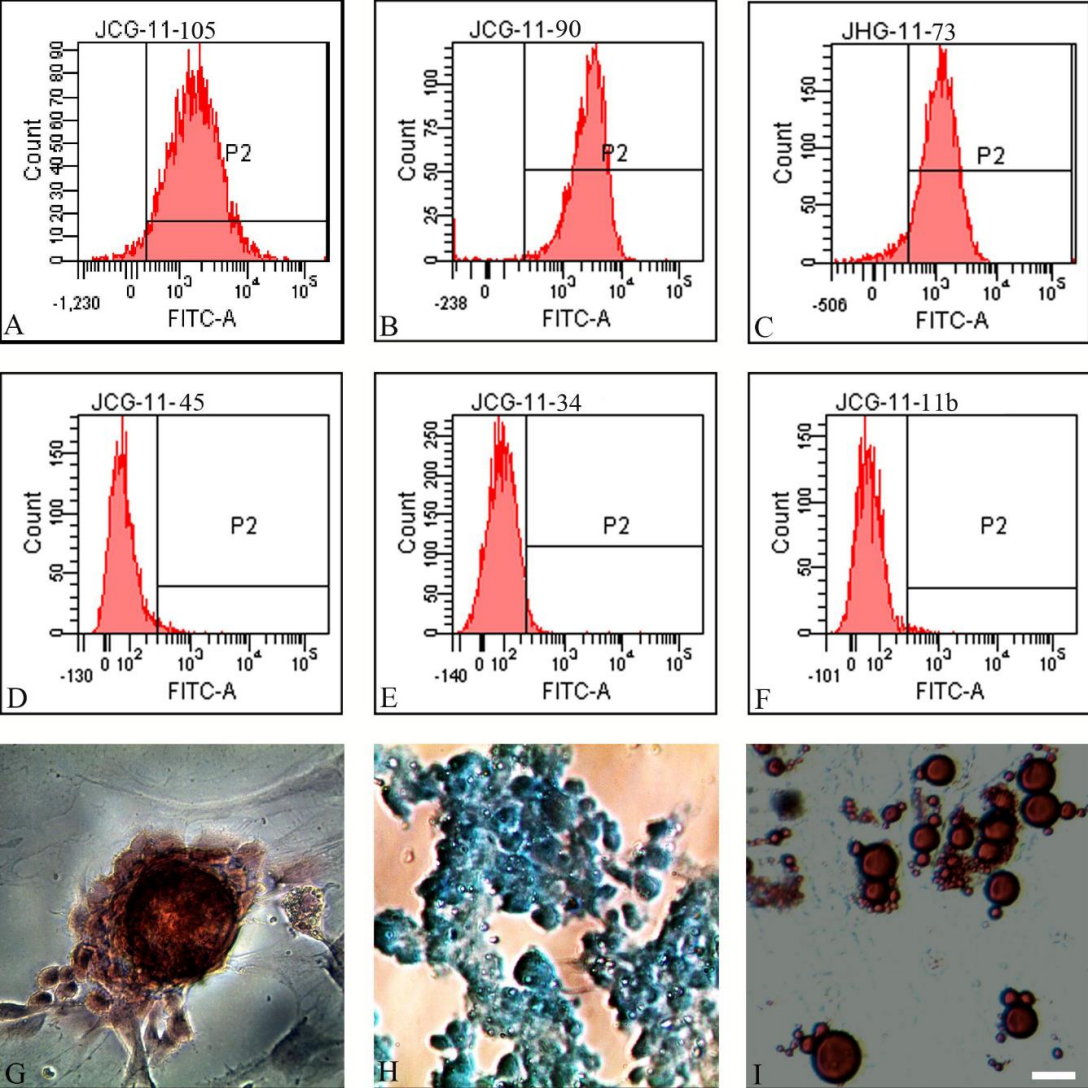
Characterization of BMSCs BMSCs were analyzed by flow cytometry for the stromal markers - CD105 (99.0%) (**A**), CD90 (98.5%) (**B**), and CD73 (96.4%) (**C**), and hematopoietic markers - CD45 (2.2%) (**D**), CD34 (0.6%) (**E**) and CD11b (3.6%) (**F**). After BMSCs were induced for Osteogenesis (3 weeks), Chondrogenesis (2 weeks), and Adipogenesis (2 weeks) using differentiation Kits, alizarin red, alcian blue and oil red O staining visualized osteoblasts (**G**) (red), chondroblasts (**H**) (blue) and adipocytes (**I**) (red), respectively. Bar = 20μm.

### RNA extraction and qRT-PCR

The expression profiles of Notch signaling and GABAergic neuron-like differentiation of BMSCs were further confirmed by qRT-PCR. Total RNA was extracted from the pre-induced cell samples or induced cell samples using Trizol® Reagent (Invitrogen, 15596-018, CA) according to the manufacturer’s instructions. Total RNA was reverse transcribed into cDNA using Moloney-murine leukemia virus reverse transcriptase and Oligo dT (Promega, WI). All the primer sequences were designed and optimized by TaKaRa (TaKaRa, Dalian, CHN) as shown in Table 1. Reverse transcription was performed using the TaqMan® MicroRNA Reverse Transcription Kit (Thermo Fisher, 4366597, MA, USA) on a QuantStudio 5 Real-Time PCR System (Thermo Fisher, MA, USA). Total RNA was used as a template in control PCR reaction and normalization was based on the GAPDH gene expression. All data were obtained from at least three independent experiments and analyzed using Bio-Rad CFX 2.1 in triplicate.

### Statistical analysis

All data are presented as the Mean ± SD. SPSS 16.0 software was used for statistical analysis. One-way ANOVA with Bonferroni and Post-hoc tests was used to analyze the data, and multiple comparisons for significance were performed using Student’s *t*-test (two-sided). A *P*-value < 0.05 was considered as statistically significant.

## RESULTS

### Characterization of BMSCs

Flow cytometry results showed that BMSCs expressed high levels of stromal markers including CD105 (99.0%) (Fig. 1A), CD90 (98.5%) (Fig. 1B), and CD73 (96.4%) (Fig. 1C), and low level of hematopoietic markers such as CD45 (2.2%) (Fig. 1D), CD34 (0.6%) (Fig. 1E) and CD11b (3.6%) (Fig. 1F). In addition, analyses of multipotency demonstrated that BMSCs differentiated into osteoblasts (Fig. 1G), chondroblasts (Fig. 1H), and adipocytes (Fig. 1I) following induction, using osteogenesis (3 weeks), chondrogenesis (2 weeks), and adipogenesis (2 weeks) differentiation kits, respectively. Together, these results confirmed that the characteristics of these cells met the specific criteria of BMSCs [33].

**Figure 2.**
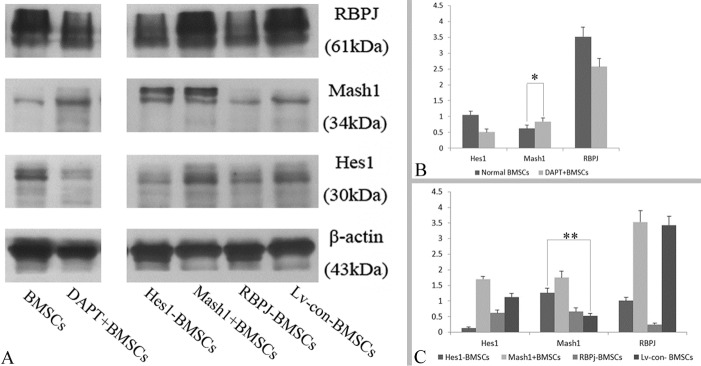
Protein level of GABAergic determinants in different manipulated BMSCs **A**) Protein bands of Notch signaling (RBPJ, Hes1 and Mash1) visualized through western blotting of BMSCs that were treated by DAPT (DAPT+BMSCs), or engineered by Hes1 shRNA (Hes1-BMSCs), pGC-FU-Mash1 plasmid (Mash1+BMSCs), RBPJ shRNA (RBPJ-BMSCs) and copGFP Control (Lv-con-BMSCs). **B**) The protein level based on the ratio of Gauss Model Trace exhibited the Notch signaling (RBPJ, Hes1 and Mash1) changes in the DAPT+ BMSCs. **C**) The protein level based on the ratio of Gauss Model Trace exhibited genetically engineered BMSCs. Error bars in bar graphs display standard deviation (SD). *P = 0.032, < 0.05, **P = 0.0006, < 0.01.

**Figure 3.**
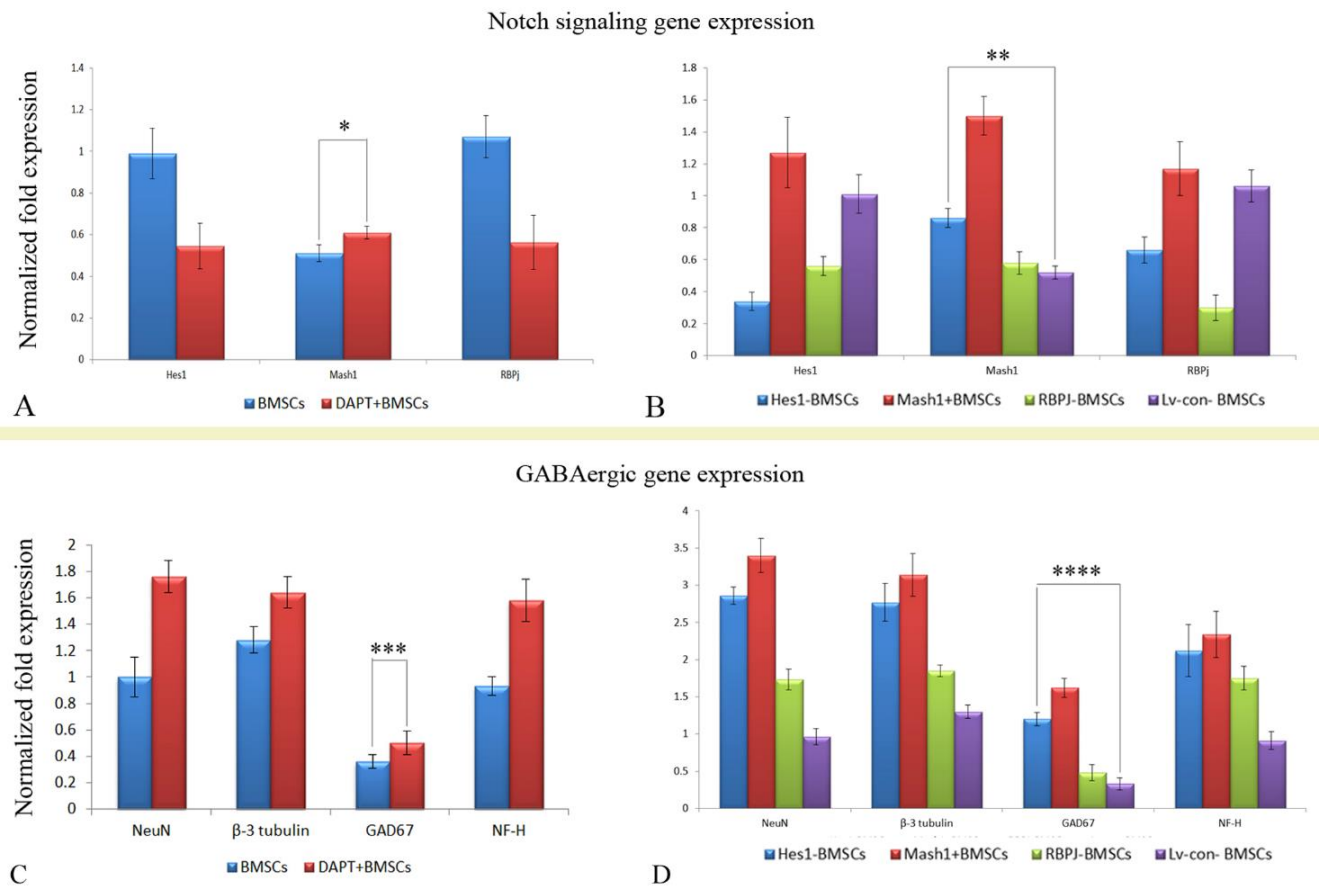
RNA level of Notch signaling and GABA-ergic gene expression in different cell samples **A**) Notch signaling gene expression in DAPT+ BMSCs. **B**) Following induction through the cocktail conditioned medium, RNA levels are shown for the neuronal and GABAergic differentiation of the DAPT+ BMSCs. **C**) Notch signaling gene expression in genetically engineered BMSCs. **D**) RNA levels are shown for genetically engineered BMSCs induced by the cocktail conditioned medium. *P = 0.027, < 0.05, **P = 0.0009, < 0.01, ***P = 0.036, < 0.05, ****P = 7.80872E-06, < 0.01.

### Effects of modulating Notch signaling pathway on the expression of Notch mediator genes in BMSCs

After BMSCs were treated with DAPT, Western blotting results (Fig. 2A) showed that the protein level (based on the ratio of Gauss Model Trace) of Notch signaling target gene - Hes1 and its effector - RBPJ were decreased (n=5/group, *P* < 0.01, vs. BMSCs), but the expression of Mash1 was increased in comparison to non-treated BMSCs (n=5/group, *P = 0.032, < 0.05; Fig. 2B). qRT-PCR further confirmed that DAPT treatment inhibited the mRNA expression of Hes1 and RBPJ (n=4/group, *P* < 0.01, vs. BMSCs), in addition to inducing an upregulation of Mash1 mRNA expression in BMSCs (n=4/group, *P = 0.027, < 0.05; Fig. 3A). Thus, treatment with a γ-secretase inhibitor (i.e. DAPT) induced repression of the Notch signaling pathway in BMSCs as expected.

To elucidate the regulatory role of Notch signaling in BMSCs, an RBPJ shRNA, Hes1 shRNA, or pGC-FU-Mash1 plasmid was used to manipulate the expression of corresponding genes. Western blotting showed that BMSCs transduced with Hes1 shRNA displayed downregulation of RBPJ (n=5/group, *P* = 0.0001, < 0.01) and upregulation of Mash1 (n=5/group, **P = 0.0006, < 0.01), in comparison to BMSCs transduced with control lentiviral vector (Lv-con-BMSCs) (Fig. 2C). After BMSCs were transduced with RBPJ shRNA, a decreased expression of Hes1 (n=4/group, *P* = 0.0002, < 0.01) was observed, but Mash1 expression was not increased significantly (n=4/group, *P* = 0.14, > 0.05) compared with Lv-con-BMSCs (Fig. 2C). Together, these results implied that a reciprocal inhibition might exist between Hes1 and RBPJ, and Hes1 gene inhibition results in the upregulation of Mash1 in BMSCs. When the BMSCs were engineered with a pGC-FU-Mash1 plasmid, Hes1 expression presented with a remarkable increase (n=4/group, *P* = 0.0004, < 0.01), but the expression of RBPJ did not increase in comparison to Lv-con-BMSCs (n=4/group, P>0.05, Fig. 2C). Thus, these results indicated that overexpression of Mash1 is capable of activating the expression of Hes1 gene, and the expression modulation between RBPJ and Mash1 is not significant in BMSCs. Meanwhile, qRT-PCR results also demonstrated that downregulation of Hes1 gene in BMSCs resulted in a decreased expression (n=4/group, *P* = 0.001, < 0.01, vs. Lv-con-BMSCs) of RBPJ and an increased expression (n=4/group, **P = 0.0009, < 0.01, vs. Lv-con-BMSCs) of Mash1, while RNA interference of RBPJ in BMSCs was accompanied with Hes1 inhibition (n=4/group, *P* = 0.002, < 0.01, vs. Lv-con-BMSCs) and overexpression of Mash1 upregulated the Hes1 expression (n=4/group, *P* = 0.043, < 0.05, vs. Lv-con-BMSCs) in BMSCs (Fig. 3B). In summary, these results indicated that Hes1 and RBPJ promote the expression of each other via the Notch signaling pathway, and Mash1 may be a compensator and activator of Hes1 expression in BMSCs.

**Figure 4.**
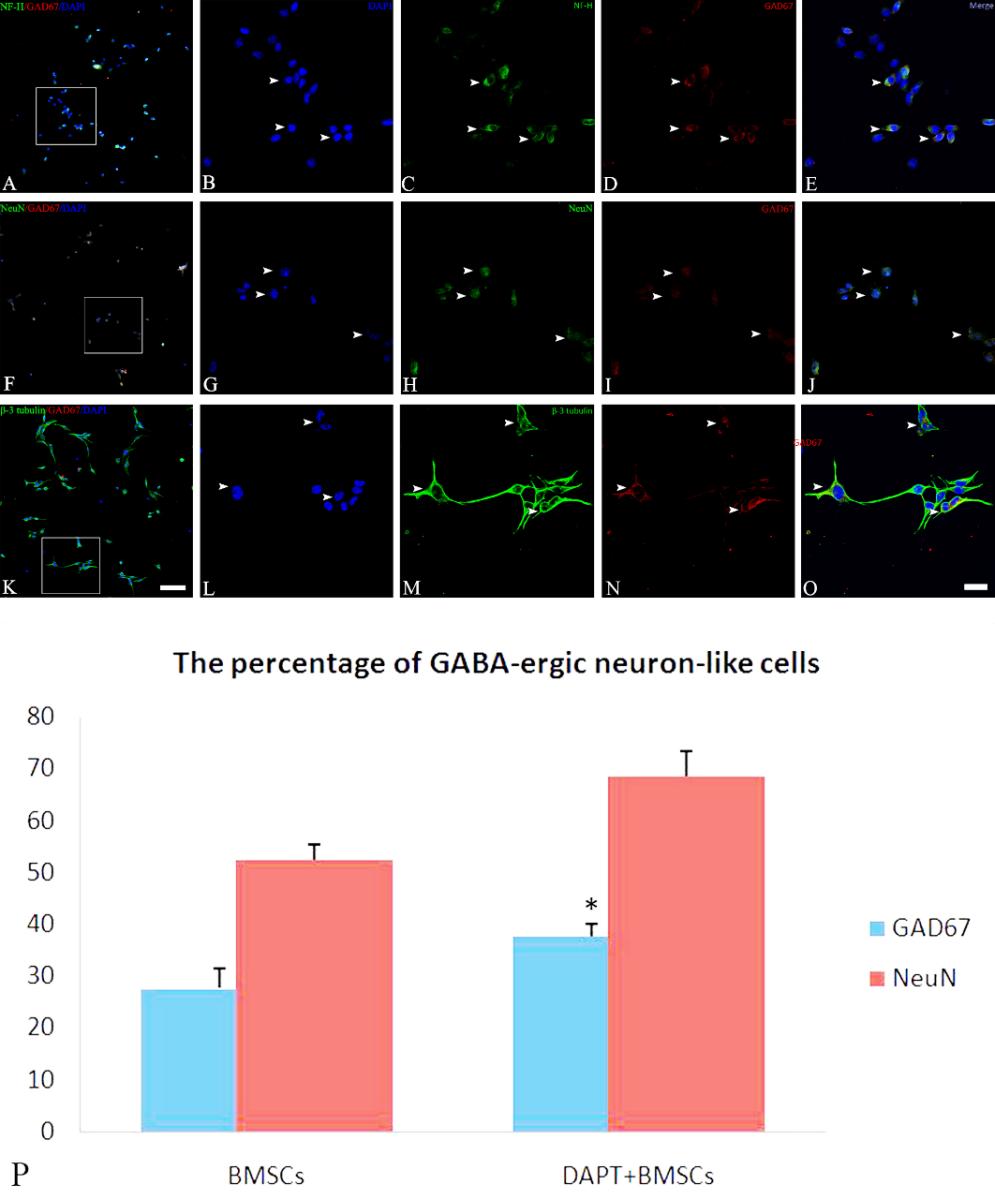
Immunofluorescence for GABA-ergic neuron-like differentiation from BMSCs After BMSCs were induced by the cocktail conditioned medium, immunofluorescence showed that differentiated cells expressed neuronal cytoskeleton marker NF-H (**C**, white arrow), mature neuronal marker NeuN (**H**, white arrow), specific neuronal marker (β-3 tubulin (**M**, white arrow), and GABA-ergic cell marker GAD67 (**D, I** and **N**, white arrow). In addition, a few GAD67 positive cells are co-localized with NF-H (**A** / **E**, white arrow), NeuN (**F**/**J**, white arrow), and β-3 tubulin (**K**/**O**, white arrow). **B, G** and **L** illustrate DAPI stained nuclei. Bar (**A, F** and **K**) = 50 μm, bar (**E, J** and **O**) = 20 μm. Statistical analysis shows the percentage of GABA-ergic neuron-like differentiation in DAPT treated BMSCs (**P**). *P = 0.002, < 0.01, vs. BMSCs.

**Figure 5.**
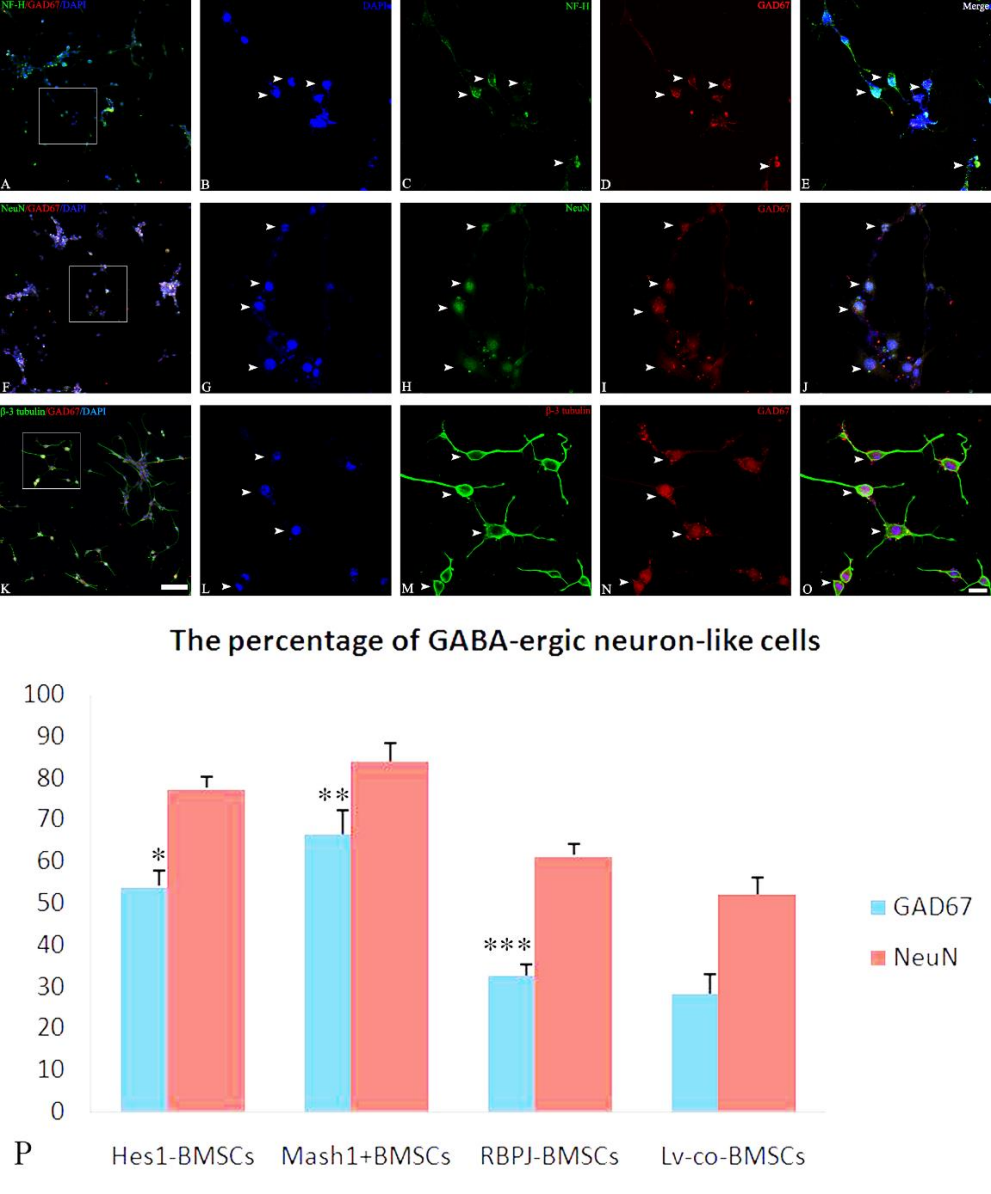
Immunofluorescence showing GABAergic neuron-like differentiation from Mash1 overexpressed BMSCs After Mash1+ BMSCs were induced by cocktail conditioned medium, immunofluorescence showed that differentiated cells expressed neuronal cytoskeleton marker NF-H (**C**, white arrow), mature neuronal marker NeuN (**H**, white arrow), and specific neuronal marker β-3 tubulin (**M**, white arrow), in addition to GABA-ergic cell marker GAD67 (**D, I** and **N**, white arrow). Note that, many GAD67 positive cells co-localize with NF-H (**A** / **E**, white arrow), NeuN (**F** / **J**, white arrow), and β-3 tubulin (**K**/**O**, white arrow). **B, G** and **L** illustrate DAPI stained nuclei. Bar (**A, F** and **K**) = 50 μm, bar (**E, J** and **O**) = 20 μm. Statistical analysis shows the percentage of GABA-ergic neuron-like differentiation in genetically engineered BMSCs (**P**). Statistical differences referred to Lv-con-BMSCs, *P = 1.9036E-05, < 0.01; **P = 3.61045E-05, < 0.01; ***P = 0.122, > 0.05.

### Effects of modulating Notch signaling pathway on GABAergic differentiation of BMSCs

Immunofluorescence staining was used to characterize the GABAergic differentiation of BMSCs. The results showed that induced BMSCs expressed the neuronal cytoskeleton marker NF-H (Fig. 4C), GABAergic cell marker GAD67 (Fig. 4D, I and N), mature neuronal marker NeuN (Fig. 4H), and a neuron-specific marker β-3 tubulin (Fig. 4M). A smaller percentage (27.6±3.85 %, n=6/group,) of cells expressed both GAD67 and NeuN (Fig. 4J and P) after induction. Moreover, the GAD67 expression was also found co-localized with other neuronal markers such as NF-H (Fig. 4E) and β-3 tubulin (Fig. 4O), which implied that BMSCs have the capability to differentiate into GABAergic neuron-like cells *in vitro*. When DAPT-treated BMSCs, Hes1-knockdown BMSCs, or Mash1-overexpressing BMSCs (Fig. 5A-O) were induced by cocktail conditioned medium, greater numbers of cells positive for both GAD67 and NeuN were observed (Fig. 5P). In addition, increased numbers of GABAergic neuron-like cells could be induced from DAPT-treated BMSCs (*P = 0.002, < 0.01, vs. BMSCs n=6/group) (Fig. 4P), Hes1- BMSCs (*P = 1.9036E-05, < 0.01, vs. Lv-con-BMSCs, n=6/group) or Mash1+ BMSCs (**P = 3.61045E-05, < 0.01, vs. Lv-con-BMSCs, n=6/group), but the difference was not significant (***P = 0.122, > 0.05) between induced RBPJ-silenced BMSCs and induced Lv-con-BMSCs (Fig. 5P). These results suggest that Notch signaling inhibition enhances the neuronal differentiation of BMSCs, and differentiation of BMSCs into GABAergic neuron-like cells is promoted by Hes1 silencing or Mash1 overexpression, but not by RBPJ silencing.

**Figure 6.**
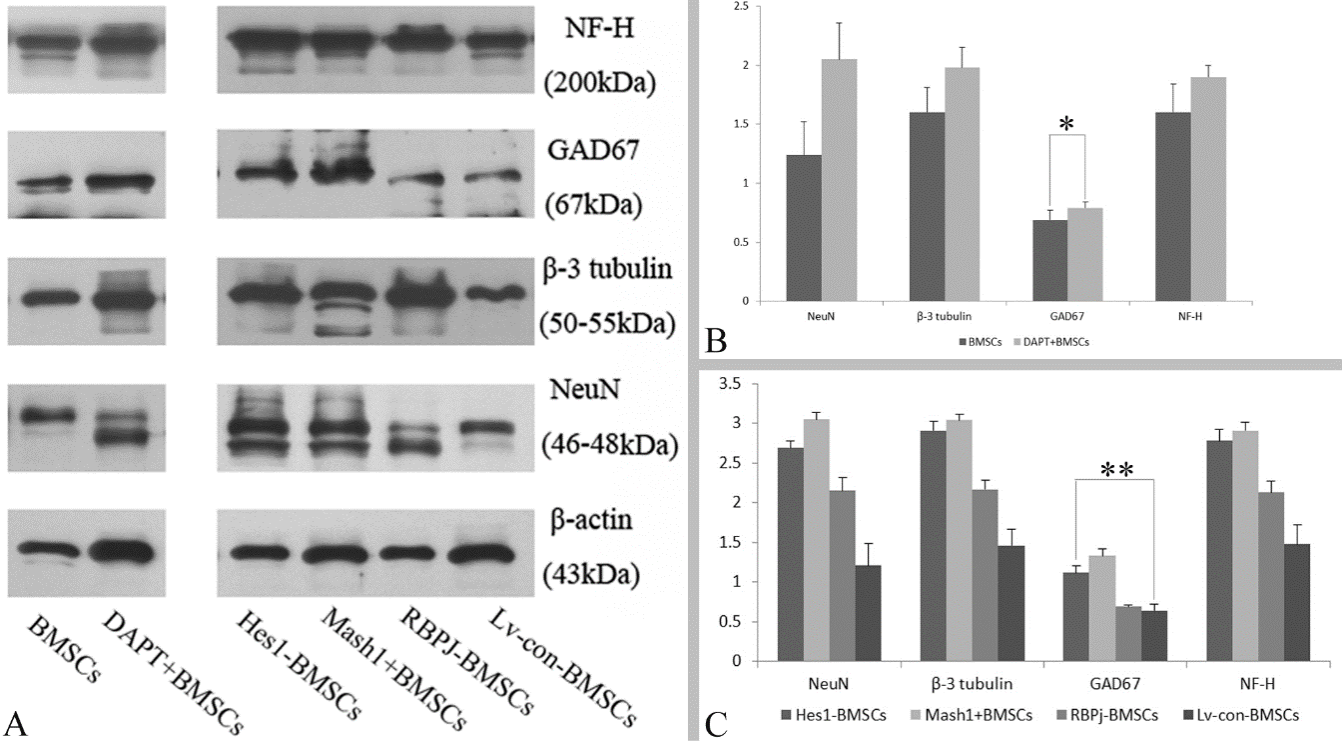
Western blotting results for the GABA-ergic differentiation of BMSCs **A)** Protein bands of neuronal (NF-H, NeuN, and β-3 tubulin) and GABA-ergic (GAD67) neuronal markers after DAPT+ BMSCs (right part) and genetically engineered BMSCs (left part) induced by cocktail medium. **B)** Protein level based on the ratio of Gauss Model Trace confirmed the neuronal (including GABA-ergic) differentiation of the DAPT+ BMSCs. **C)** Protein level based on the ratio of Gauss Model Trace further confirmed the neuronal (including GABA-ergic) differentiation of the genetically engineered BMSCs. *P = 0.025, < 0.05, **P = 0.0005, < 0.01.

Western blottingand qRT-PCR (Fig. 3C and D and Fig. 6) were also employed to quantitatively characterize the capability of different cell samples to differentiate into GABAergic neuron-like cells. One-way ANOVA analysis demonstrated that the protein (Fig. 6B) and RNA levels (Fig. 3C) of the neuronal (NeuN) or GABAergic (GAD67) marker expression were increased after induction in DAPT-treated BMSCs in comparison to non-treated BMSCs (*P* < 0.05, n=5/group). In addition, DAPT treatment increased the expression of neuronal markers including NF-H and β-3 tubulin at both protein (Fig. 6B) and mRNA levels (*P* < 0.05, n=5/group) (Fig. 3C), further confirming that the inhibition of the Notch signaling pathway promotes GABAergic differentiation of BMSCs. On the other hand, when genetically engineered BMSCs (Hes1-BMSCs, Mash1+BMSC or RBPJ-BMSCs) were induced by the cocktail conditioned medium, protein (Fig. 6C) and mRNA measurements (Fig. 3D) showed an increased expression of neuronal markers (NF-H, NeuN and β-3 tubulin, *P* < 0.05, vs. induced Lv-con-BMSCs, n=4/group). An increased level of GAD67 expression (*P* < 0.01, vs. induced Lv-con-BMSCs, n=4/group) was also seen in the induced Mash1-overexpressing BMSCs and Hes1-silenced BMSCs, but not in RBPJ-silenced BMSCs (*P* > 0.05, vs. induced Lv-con-BMSCs, n=4/group) (Fig. 6C and Fig. 3D). Collectively, these results imply that the upregulation of the Mash1 gene is required for promoting GABAergic differentiation of BMSCs.

## DISCUSSION

Several studies have showed differentiation of BMSCs into GABAergic neuron-like cells to facilitate the use of an autologous source of donor cells for grafting in neurological disorders. Some studies have used beta-mercaptoethanol and retinoic acid (RA) as pre-inducers followed by potassium chloride [34], and others have employed differentiation cocktails comprising a hippocampal astrocyte and glioblastoma conditioned medium [7] or conditioned medium including Bt2cAMP and acid ATRA [11]. Moreover, our previous study demonstrated that overexpression of Mash1 can enhance the differentiation of BMSCs into functional GABAergic neuron-like cells [11]. Nonetheless, a method that greatly enhances the production of GABAergic neuron-like cells from BMSCs is yet to be discovered. While it is well known that the Notch signaling is an evolutionarily conserved system that regulates proliferation and differentiation of BMSCs [29, 30], the regulatory role of Notch signaling in the differentiation of BMSCs into GABAergic neuron-like cells has not been examined.

In this study, we sought to elucidate the mechanisms by which Notch signaling participates in the differentiation of BMSCs into GABAergic cells. We first examined the effect of inhibition of Notch signaling through γ-secretase inhibitor DAPT on differentiation of BMSCs into GABAergic neuron-like cells. Our results showed that the Notch signaling target gene (Hes1) and its effector (RBPJ) displayed a significant decrease, while the Notch signaling downstream gene (Mash1) exhibited an increase following DAPT treatment in BMSCs. Inhibition of γ-secretase by DAPT prevents the release of NICD, translocation of NICD to the nucleus, and NCID binding to RBPJ. This intervention, in turn, prevents the expression of downstream target gene Hes1 [22, 31] and results in the elevation of Mash1 expression in the canonical Notch signaling pathway [20]. These results suggest that DAPT treatment inhibits the Notch signaling pathway of BMSCs as seen in neural stem cells earlier. Furthermore, an increased expression of the standard neuronal markers and GABAergic neuronal markers was observed when DAPT-treated BMSCs were induced through the cocktail conditioned medium, which implies that inhibition of the Notch signaling pathway promotes not only neuronal differentiation but also the specific transformation of BMSCs into GABA+ neuron-like cells.

Next, we evaluated the regulatory role of Notch signaling in BMSCs by genetic engineering, as it has been suggested that the Notch pathway participates in the GABAergic differentiation of BMSCs through changes in RBPJ, Hes1 and Mash1 signaling [11, 21, 28]. Specifically, we found that BMSCs treated with RBPJ shRNA or Hes1 shRNA displayed a decreased expression of Hes1 or RBPJ. Furthermore, an increased expression of Mash1 was observed in BMSCs treated with Hes1 shRNA but not in BMSCs treated with RBPJ shRNA. Based upon this, it appeared that RBPJ and Hes1 activate the expression of each other in the Notch signaling pathway, and as a result, Mash1 can be up regulated with Hes1 repression in BMSCs. In consideration to the expression of genes in DAPT-treated BMSCs, the results might suggest that a reciprocal inhibition between Hes1 and RBPJ exists in BMSCs, and the Mash1 gene plays a compensatory role when Hes1 is downregulated in BMSCs. Previous studies have indicated that the Hes1 gene is essential for controlling the number and diversity of stem cells as well as antagonizing activator-type bHLH proneural genes such as Mash1. Upregulation of the Mash1 gene in stem cells also induces the expression of Notch ligands such as Delta to activate Notch signaling, thereby maintaining stem cells in an undifferentiated state [20, 25]. Accordingly, our results showed that Hes1 signaling increased after Mash1 overexpression in BMSCs, showing that Mash1 can also activate the Notch signaling target gene Hes1 in BMSCs. Silencing of RBPJ, though inhibited Hes1, had no impact on Mash1 expression. In addition, Mash1 overexpression did not influence RBPJ expression in BMSCs. Generally, studies using neural stem cells have demonstrated that RBPJ silencing results in the down regulation of Mash1, while upregulation of Mash1 activates RBPJ expression in the canonical Notch signaling pathway [20]. However, it is likely that, in addition to Notch signaling, stem cell fate decision and multiple biological processes are controlled by many signaling pathways [35-37]. From this perspective, our results may suggest that the regulatory role between RBPJ and Mash1 in BMSCs involves other mechanisms, which need to be examined in future studies.

We also analyzed the influence of Notch signaling on GABAergic neuron-like differentiation. The overall differentiation of BMSCs into neuron-like cells was increased by inhibiting Notch signaling (such as repressing γ-secretase or knocking down Hes1) or overexpressing Mash1. This finding revealed that manipulation of Notch signaling could enhance the neuronal differentiation of BMSCs under the conditioned induction, which is consistent with the neuronal commitment mechanisms seen in neural stem cells [20, 27, 38]. Although the role of Notch signaling (such as Notch1, Hes1, Jagged-1, NICD, Hey-1, etc.) in determining neuronal fate has been studied in MSCs earlier [23, 29], its regulatory role in the differentiation of BMSCs into GABAergic neuron-like cells was unknown. Our current results demonstrate that differentiation of BMSCs into GABAergic neuron-like cells can be enhanced by knocking down Hes1 or overexpressing Mash1, but not by knocking down RBPJ. Furthermore, upregulation of the Mash1 gene was observed in Hes1-knockdown BMSCs but not in RBPJ-knockdown BMSCs despite lower Hes1 expression in these cells. It has been reported that transactivation activity of Mash1 is required for the production of neocortical GABAergic neurons and decrease of Mash1 gene expression results in a complete loss of the GABAergic phenotype [19, 39]. Additionally, the Mash1 gene has been considered important for determining GABAergic neuronal specification [27]. Thus, increased GABAergic neuron-like differentiation from DAPT-treated BMSCs is accompanied by Mash1 upregulation, which implies that the Mash1 gene determines the GABAergic differentiation of BMSCs under the conditioned induction as well.

### Conclusions

Mash1-dependent Notch signaling regulates the differentiation of BMSCs into GABAergic neuron-like cells. GABAergic neuron-like cells derived from genetically engineered BMSCs may be a promising source for cell therapy in neurological disorders.
